# Decision Making in the Reward and Punishment Variants of the Iowa Gambling Task: Evidence of “Foresight” or “Framing”?

**DOI:** 10.3389/fnins.2012.00107

**Published:** 2012-07-19

**Authors:** Varsha Singh, Azizuddin Khan

**Affiliations:** ^1^Department of Organizational Behavior and Human Resources, Indian Institute of ManagementKozhikode, India; ^2^Department of Management, Indian Institute of ScienceBangalore, India; ^3^Department of Psycho-physiology laboratory, Humanities and Social Sciences, Indian Institute of TechnologyMumbai, India

**Keywords:** Iowa gambling task, reward–punishment, instructions, decision making, framing effect

## Abstract

Surface-level differences in the reward and punishment variants, specifically greater long-term decision making in the punishment variant of the Iowa Gambling Task (IGT) observed in previous studies led to the present comparison of long-term decision making in the two IGT variants (*n* = 320, male = 160). It was contended that risk aversion triggered by a positive frame of the reward variant and risk seeking triggered by a negative frame of the punishment variant appears as long-term decision making in the two IGT variants. Apart from the frame of the variant as a within-subjects factor (variant type: reward and punishment), the order in which the frame was triggered (order type: reward–punishment or punishment–reward), and the four types of instructions that delineated motivation toward reward from that of punishment (reward, punishment, reward and punishment, and no-hint) were hypothesized to have an effect on foresighted decision making in the IGT. As expected, long-term decision making differed across the two IGT variants suggesting that the frame of the variant has an effect on long-term decision making in the IGT (*p* < 0.001). The order in which a variant was presented, and the type of the instructions that were used both had an effect on long-term decision making in the two IGT variants (*p* < 0.05). A *post hoc* test suggested that the instructions that differentiated between reward and punishment resulted in greater foresight than the commonly used IGT instructions that fail to distinguish between reward and punishment. As observed in previous studies, there were more number of participants (60%) who showed greater foresight in the punishment variant than in the reward variant (*p* < 0.001). The results suggest that foresight in IGT decision making is sensitive to reward and punishment frame in an asymmetric manner, an observation that is aligned with the behavioral decision making framework. Benefits of integrating findings from behavioral studies in decision neuroscience are discussed, and a need to investigate cultural differences in the IGT studies is pointed out.

## Introduction

The somatic marker hypothesis (SMH) states that emotions are indispensible to long-term decision making (Damasio, 1994). Support for the hypothesis comes from observing healthy participants’ ability to make long-term advantageous decisions on a task called the Iowa gambling task (IGT; Bechara et al., 1994). In order to rule out reward and punishment sensitivity as an alternative explanation for decision making on the task, Bechara et al. (2000b) compared reward and punishment variants of the IGT to demonstrate long-term advantageous decision making irrespective of the immediate reward and punishment frame of the IGT. However, in the most examined reward variant, the magnitude (Tomb et al., 2002; van den Bos et al., 2006) and frequency of immediate reward and punishment (Chiu and Lin, 2007; Lin et al., 2007; Chiu et al., 2008) continue to confound long-term decision making in the IGT.

In the current paper, the effect of reward and punishment sensitivity on long-term decision making in the two variants is examined. Three observations have led to the current examination of the two variants. (1) In the original and subsequent studies, there are on-the-surface differences in long-term decision making in the two variants, such that higher long-term advantageous decision making is seen in the punishment variant (e.g., Bechara et al., 2000b, 2002; Must et al., 2006, 2007; Verdejo-Garcia et al., 2006). (2) Differences in long-term decision making in the two variants might be masked by using an unequal criterion for judging impairment in the two variants (i.e., a score less than 10 in the reward variant and less than 8 in the punishment variant; Bechara et al., 2002). Unequal cut-off criteria suggest a difference in the ability to make long-term advantageous decisions in the two variants. (3) Judging by the direction of inequality in the cut-off scores, long-term decision making in the punishment variant seems more difficult. However, more number of healthy participants were “impaired” in the reward variant and “unimpaired” in the punishment variant (56%), whereas only a small number of participants (4.5%) showed the opposite trend (Bechara et al., 2002), suggesting greater difficulty in making long-term decision making in the reward variant.

A difference in long-term decision making in the two variants is expected based on the following extrapolation:

(1) Even though both the variants contain rewards and punishments the reward variant triggers a positive frame and the punishment variant triggers a negative frame. A brief description of the two variants will be helpful in understanding how the immediate “frame” of the variant might affect long-term decision making on the IGT. The reward variant offers a choice between four decks of cards labeled A′, B′, C′, and D′. The participant has to pick one card at a time; after a card is picked, an announcement of the amount “won” is flashed on the computer screen, occasionally followed by an announcement of a “loss.” The punishment variant offers a choice between four decks of cards labeled E′, F′, G′, and H′. After a card is picked, the “loss” is announced, which at times is followed by a “gain.” Therefore in spite of both the variants offering both, rewards and punishments, the prominent outcome in the reward variant is a “win,” and in the punishment variant a “loss,” which underlies the assertion that a positive frame (i.e., “gain”) is triggered in the reward variant and a negative frame (i.e., “loss”) is triggered in the punishment variant. Unknown to the decision maker, decks A′ and B′ have high immediate rewards and a net loss, while decks C′ and D′ have small immediate rewards and a net gain. Long-term advantageous decision making is reflected in avoiding the risky decks (decks A′ and B′) and seeking the safe decks (decks C′ and D′). In the punishment variant, decks F′ and H′ give immediate low losses and a low net gain, while decks E′ and G′ give immediate high losses and a high net gain. Long-term advantageous decision making is reflected in choosing high immediate punishment decks (decks E′ and G′) and avoiding low-immediate-punishment decks.
(2) The dominant behavioral response required for long-term decision making in the positive frame of the reward variant is avoidance of the risky decks (decks A′ and B′), and in the negative frame of the punishment variant, seeking of the risky decks or endurance of high immediate punishments (decks E′ and G′). It is possible that in the previous studies, risk aversion triggered in the reward variant resulted in safe choices (i.e., choice of decks C′ and D′) and risk taking triggered in the punishment variant resulted in choice of risky high immediate punishment (i.e., choice of decks E′ and G′), choice in both the variants appearing as long-term advantageous decision making. Therefore it is contended that long-term decision making in the two variants might demonstrate a “framing” effect (Tversky and Kahneman, 1981) rather than “foresight” and its immunity to reward–punishment sensitivity (Bechara et al., 1994).

The first step in testing the effects of reward and punishment frame in the IGT decision making taken was to test the effect of the variant type (i.e., reward and punishment frames of the IGT), and address a methodological problem that was observed in the previous studies, i.e., lack of a counter-balanced presentation of the variants (e.g., Bechara et al., 2000b, 2002; Verdejo-Garcia et al., 2006). The effect of the order in which the variant is presented would further indicate a “framing” effect suggesting that the order in which a frame is triggered also has an impact on foresighted decision making in the IGT.

To attribute the effect of variant type and order type to reward and punishment sensitivity, task motivation toward reward and punishment was altered via task instructions. Commonly used instructions for both the variants (henceforth standard instructions) are bi-directional (i.e., the decision maker is asked to seek rewards as well as avoid punishments; Bechara et al., 1994) and trigger sensitivity to both reward (gain), and punishment (loss). The standard instructions assume that long-term decision making is indifferent to reward and punishment (i.e., the decision maker is equally motivated to seek rewards and to avoid punishments). However, the standard instructions are known to convey risk-avoiding clues on which long-term decision making in the reward variant was dependent (Blair and Cipolotti, 2000; Balodis et al., 2006; Fernie and Tunney, 2006). It is possible that the only part of the standard instructions that directs one to avoid punishment is attended in the reward variant which would be compatible with the framing effect explanation. The uni-directional instructions (i.e., the decision maker is motivated either to seek rewards or to avoid punishments) will delineate sensitivity to rewards from that of punishment, and the effect of instruction alteration on long-term decision making in the two variants will indicate a pronounced framing effect or the effect of reward and punishment. In line with the assertion that reward and punishment sensitivity has an effect on IGT decision making, it was hypothesized that variant, order, and instruction types will have an effect on long-term decision making in the IGT.

## Materials and Methods

### Sample

Three-hundred twenty healthy undergraduate and graduate students volunteered for the study (mean age = 23.82; SD = 3.25 years; male = 160). All the participants had more than 18 years of education (22.7% were enrolled in a bachelor’s program, 44.9% were enrolled in a master’s program, and 32.4% were enrolled in a doctoral program). Most of the participants were right handed (86.1%) and non-smokers (93.6%). All the participants were medication-free, had never experienced a head injury that required hospitalization, and had never been diagnosed with a psychiatric illness.

### Design

A 2 (variant order: reward–punishment and punishment–reward) × 2 (variant type: total net score on reward and total net score on punishment variant) × 4 (instruction type: avoid punishment, seek reward, standard, and no-hint) design was used in the study. Within-subject variables were total net scores on the reward variant and the punishment variant, and between subject variables were type of order and type of task instruction used.

Decision making in the variants was analyzed according to the “net score” method (Bechara et al., 1994), that is, the number of cards drawn from decks A′ and B′ are added and their sum is deducted from the number of cards drawn from decks C′ and D′ [(decks C′ + D′) − (decks A′ + B′)]. This is done for a block of 20 trials each, and scores on 5 blocks are added to get a total net score in the reward variant. In the punishment variant, the formula is [(E′ + G′] − (F′ + H′)] for five blocks of trials added to get a total net score.

### Materials

The computerized IGT progressive reward variant (A′B′C′D′) and progressive punishment variant (E′F′G′H′) were used. The progressive variant is slightly different from the original IGT because it exaggerates the future outcome, that is, it increases the magnitude of long-term rewards in the advantageous decks and the long-term punishments in the risky decks (Bechara et al., 2000b). Four types of instructions were used with suitable changes to the original (standard) IGT instructions (see Appendix).

### Procedure

**Figure 1:**
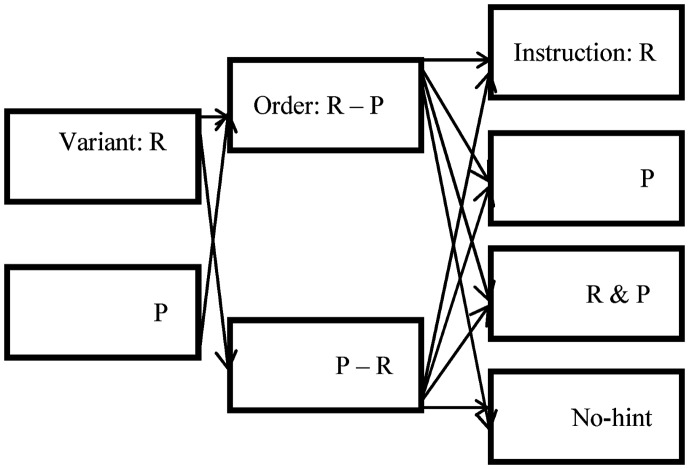
**Diagram showing variant type as a within-subjects factor (R = reward variant and P = punishment variant), order type (R–P = reward variant followed by punishment variant, P–R = punishment variant followed by reward variant), and instruction type (R = seek reward, P = avoid punishment, R and P = standard IGT instruction to seek reward and avoid punishment, No-hint = no-hint of reward or punishment) as between subjects factors (*n* = 320)**.

Participants filled in demographic details in a form, were given an overview of the experiment, and provided informed consent. The study had the approval of a thesis committee (Research Progress Committee), a departmental committee, and an institute-level committee in charge of overseeing the post-graduate research program at the institute. Participants were tested individually in a laboratory and were assigned to one of the experimental conditions. Two IGT variants were presented in a counter-balanced design (i.e., reward variant followed by punishment variant or vice versa) with one of the four types of instructions (see Figure 1). Instructions were read before the first variant was presented. After finishing the first variant, a small break was given (5 min), and instructions were read for the second variant after which the second variant was presented. After completing both variants, participants were debriefed and thanked for their participation in the study.

## Results

**Figure 2:**
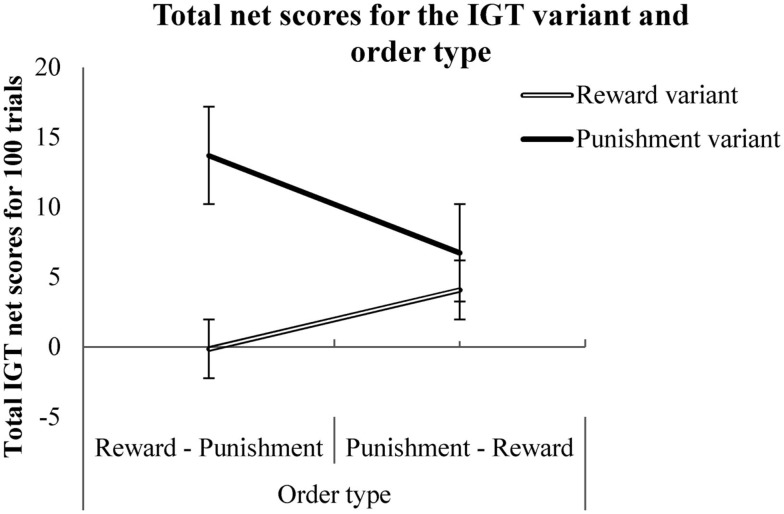
**Total net scores on the reward and the punishment variants (within-subjects) with the order of task presentation (between subjects)**. Error bars represent standard error.

**Figure 3:**
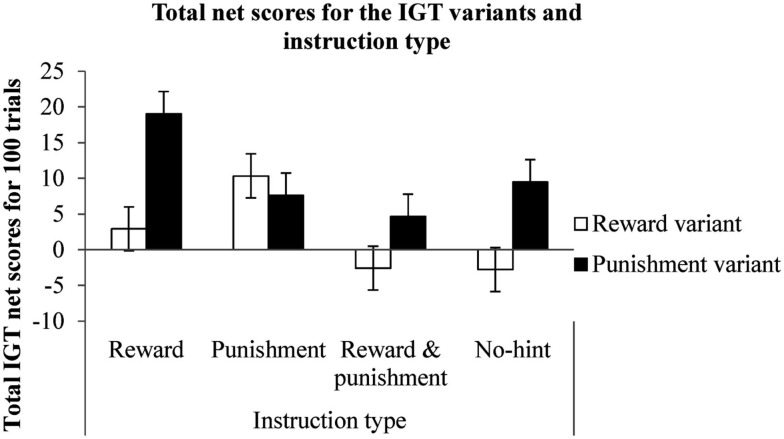
**Total net scores on the reward and the punishment variants (within-subjects) and the type of instructions (between subjects)**. Error bars represent standard error.

A mixed between-within subjects analysis of variance (ANOVA) was conducted to test the effects of order type (reward followed by punishment or punishment followed by reward variants), instruction type (seek reward, avoid punishment, standard, and no-hint) and IGT variant type (total net scores on reward and punishment variants). There was a significant within-subjects effect of variant type [Wilk’s lambda = 0.96, *F*(1, 312) = 14.66, *p* < 0.001, partial eta squared = 0.04]. The interaction of order and variant types was significant [Wilk’s lambda = 0.98, *F*(1, 312) = 3.58, *p* < 0.05, partial eta squared = 0.02; see Figure 2]. There was a significant interaction between instruction type and variant type [Wilk’s lambda = 0.97, *F*(3, 312) = 3.58, *p* < 0.05, partial eta squared = 0.03; see Figure 3]. A Tukey’s honestly significant difference (HSD) *post hoc* test showed that the instructions to seek reward had resulted in significantly higher IGT net scores compared to the standard IGT instructions (*p* < 0.05). A two-tailed binomial test showed that the number of participants making more advantageous decisions in the punishment variant than in the reward variant was greater irrespective of order or instruction type (*p* < 0.001; see Table 1).

**Table 1:** **Mean and standard deviations of total net scores in the two variants by order (*n* = 160; male = 80) and instruction types (*n* = 40; male = 20)**.

Order type	Instruction type	Total net scores on reward variant	Total net scores on punishment variant
Reward–punishment variant	Seek reward	−01.85 (27.08)	29.10 (32.05)
	Avoid punishment	12.73 (27.09)	11.50 (36.44)
	Standard IGT	−07.35 (20.37)	03.20 (37.17)
	No-hint	−04.05 (24.43)	11.00 (23.70)
	Total	−00.13 (25.83)	13.70 (33.83)
Punishment–reward variant	Seek reward	07.70 (33.76)	09.00 (37.61)
	Avoid punishment	07.95 (26.22)	03.80 (21.65)
	Standard IGT	02.20 (26.34)	06.15 (30.73)
	No-hint	−01.50 (19.77)	08.00 (19.60)
	Total	04.09 (27.02)	06.74 (28.14)

## Discussion

Contrary to SMH postulations (Bechara et al., 2002), foresighted decision making varied across the reward and punishment variants of the IGT. An inability to make equally foresighted decisions in the two variants indicate that IGT decision making is affected by variant type (i.e., by the immediate reward or punishment frame of a decision). The impression that normal healthy adults make foresighted decisions irrespective of the variant type might be the effect of the variant, which is risk aversion in the reward variant and risk seeking in the punishment variant, masked by how the decision making in the variants is analyzed and reported (i.e., by judging impairment in the two variants using unequal cut-off scores). In an earlier study, positive and negative frame introduced prior to the reward variant of the IGT led to a frame-appropriate response (i.e., positive frame resulting in risk aversion and negative frame resulting in risk seeking) which was attributed to a spontaneous or an automatic process (Franken et al., 2006).

Consistent with the framing effect explanation for long-term decision making in the IGT, the order in which the variants were presented had an effect on the long-term advantageous decision making. It was difficult to examine on-the-surface differences between the two variants from the results of the previous studies due to an absence of a counter-balanced presentation of the two variants (e.g., Bechara et al., 2000b, 2002; Verdejo-Garcia et al., 2006) or an absence of a comparable healthy control group (Must et al., 2006, 2007). The present results point out a difference in long-term decision making in the reward and punishment variants, ruling out methodological issues.

The results showed that the alteration of reward and punishment sensitivity via task instruction had an effect on long-term decision making in the two variants. Uni-directional instructions (i.e., those that differentiated between reward and punishment) resulted in greater long-term decision making compared to the bi-directional standard instructions (i.e., those that had an indifference toward reward and punishment). This result might explain why standard instructions in the reward variant are known to encourage risk/loss avoidance more than they encourage reward-seeking (Balodis et al., 2006). It is possible that due to a positive frame imposed by the reward variant only the part of the standard instructions that directs the decision maker to avoid punishments is attended in the reward variant. In line with this assertion, it was found that delineating between the positive and negative frames of the standard instructions resulted in higher long-term decision making than the standard instructions that fail to differentiate between reward and punishment (Krawitz et al., 2010). In speculation, the right hemispheric dominance observed in the reward variant of the IGT (e.g., Bechara et al., 2000a; Manes et al., 2002; Clark et al., 2003; Bolla et al., 2004; Bark et al., 2005) might be indicative of a sensitivity to punishment and risk aversion because the right hemisphere is sensitive to negative affect (Sutton and Davidson, 1997; Davidson, 2004) and associated with risk aversion (Drake, 1985, 2002; Drake and Ulrich, 1992). Future studies could examine the right hemispheric dominance in the reward variant to determine if it indicates risk aversion or loss aversion and whether the punishment variant shows similar hemispheric activity.

The on-the-surface difference of greater long-term advantageous decision making in the punishment variant observed in the original study (Bechara et al., 2002) had led to the present investigation. As suspected, the number of participants making more long-term advantageous decisions in the punishment variant was higher (more than 60%) than in the reward variant. The results point out a difference in long-term decision making in the reward and punishment variants, contradicting the claim that IGT decision making is immune to reward and punishment orientation (Bechara et al., 1994, 2000b). The role of rewards and punishments has been a contentious issue in IGT studies. For example, contrary to the SMH-IGT assumption, the learning of rewards and punishments (Rolls et al., 1994), knowledge of rewards and punishments (Maia and McClelland, 2004), immediate rewards and punishments (van den Bos et al., 2006), and frequency of immediate rewards and punishments (Chiu and Lin, 2007; Lin et al., 2007; Chiu et al., 2008) are believed to confound long-term decision making in the reward variant of the IGT, weakening the assertion that IGT decision making is immune to reward and punishment sensitivity. The present results obtained from comparing both the variants of the IGT suggest that reward and punishment has an effect on long-term decision making in the IGT in the form of the variant type (reward and punishment), order type (reward followed by punishment and vice versa), and instruction type (either approach reward or avoid punishment, and approach reward while avoiding punishment).

There was evidence from the psychology literature that punishment or negative stimuli is potent (Kanhouse and Hanson, 1972), processed preferentially (Hansen and Hansen, 1988; Pratto and John, 1991; Lane et al., 1997), produces a strengthened response on the cognitive, emotional, and physiological levels (Taylor, 1991), and results in a stronger motivation (Taylor, 1991; Cacioppo et al., 1999) than reward or positive stimuli. It had been pointed out in the behavioral decision making literature that reward–punishment are unequally valued and have an asymmetrical influence (Tversky and Kahneman, 1981, 1991). Incorporating other relevant findings from behavioral studies such as temporal discounting, a preference for immediate reward over delayed ones (Ainslie, 1975), myopic loss aversion, an over-sensitivity to losses combined with shortsightedness (Benartzi and Thaler, 1995), preference based on frequency of reward (Loewenstein and Prelec, 1992), and on punishment (Bateman et al., 2007), will add valuable insights to a developing field of decision neuroscience.

The results underscore the role of socio-economic and cultural factors in understanding decision making in the IGT. Inconsistent with the IGT assumptions, frequencies of immediate reward and punishment rather than the inter-temporal nature of choices were determinants of IGT decision making in Taiwan (Chiu and Lin, 2007, Lin et al., 2007; Chiu et al., 2008), Iran (Ekhtiari et al., 2009), and Brazil (Bakos et al., 2010). While it is assumed that risk is perceived in terms of inter-temporality and risky decision making is manifested in the tradeoff between an immediate versus a delayed outcome (irrespective of reward or punishment as an outcome) in the IGT, socio-economic, and cultural differences in the IGT suggest an alternative definition of risk and risky decision making in the IGT. When socio-economic and cultural differences are investigated as a part of the decision neuroscience studies, it would benefit areas such as cultural neuroscience, and social neuroscience, by helping us understand the link between culture-specific decision making behavior and brain functioning.

The results pointed out a “negativity bias” in IGT decision making. Is it easier to make long-term advantageous decisions when the predominant outcome of every choice is a “loss”? Future investigations could examine the reason for the pronounced effect of a loss frame that instigates risk taking than of a gain frame that triggers risk aversion. In a task that is different from the IGT risk aversion was observed to be detrimental to long-term advantageous decision making (Shiv et al., 2005), whereas in the IGT (reward variant), risk aversion is necessary for long-term advantageous decision making (Balodis et al., 2006; Franken et al., 2006). Until now, the two variants have never been compared to test risk aversion in the reward variant and risk seeking in the punishment variant. Future studies could compare the two variants to test whether a pronounced effect of the punishment variant and risk seeking is specific to a socio-economic and cultural context. The methodology problem of counterbalancing the presentation of variants that occurred in earlier studies was addressed in this study, but the results need to be interpreted considering the limitation that the participants did not play using real money, which could be an important factor when comparing risk taking in the reward and punishment variants of IGT.

## Conflict of Interest Statement

The authors declare that the research was conducted in the absence of any commercial or financial relationships that could be construed as a potential conflict of interest.

## Appendix

Four types of instructions were used in the study: standard (1a and 1b), seek reward (2), avoid punishment (3), and no-hint (4a and 4b) instructions.

1a. Standard instructions, reward variant: “In front of you on the screen, there are four decks of cards: A′, B′, C′, and D′. When we begin the game, I want you to select one card at a time by clicking on a card from any deck. Each time you select a card, the computer will tell you that you won some money. I do not know how much money you will win. You will find this out as you go along. Every time you win, the green bar at the top of the screen gets bigger. Every so often, when you click on a card, the computer will tell you that you won some money as usual, but it will also say that you lost some money as well. I do not know when you will lose or by how much. You will find out as you go along. Every time you lose, the green bar at the top of the screen gets smaller. You are absolutely free to switch from one deck to another at any time, and as often as you wish. *The goal of the game is to win as much money as possible and to avoid losing as much money as possible*. You would not know when the game will end. Simply keep on playing until the computer stops. You will have $2,000 of credit, shown by the green bar, at the start of the game. The only hint I can give you, which is the most important thing to note, is this: Out of these four decks of cards, some are worse than others. To win, you should try to stay away from bad decks. No matter how much you find yourself losing, you can still win the game if you avoid the bad decks. Moreover, the computer does not change the position of the decks once the game begins. It does not make you lose at random, or make you lose money based on the last card you picked.”
1b. Standard instructions, punishment variant: “In front of you on the screen, there are four decks of cards: E′, F′, G′, and H′. When we begin the game, I want you to select one card at a time by clicking on a card from any deck. Each time you select a card, the computer will tell you that you lost some money. I do not know how much money you will lose. You will find this out as you go along. Every time you lose, the green bar at the top of the screen gets smaller. Every so often, when you click on a card, the computer will tell you that you lost some money as usual, but it will say that you gained some money as well. I do not know when you will gain or by how much. You will find out as you go along. Every time you gain some money, the green bar at the top of the screen gets bigger. You are absolutely free to switch from one deck to the other at any time, and as often as you wish. *The goal of the game is to avoid losing as much money as possible and to win as much money as possible*. You would not know when the game will end. Simply keep on playing until the computer stops. You will have $2,000 of credit, shown by the green bar, at the start of the game. The only hint I can give you, which is the most important thing to note, is this: Out of these four decks of cards, some are better than others. To win, you should try to choose from the good decks. No matter how much you find yourself losing, you can still win the game if you choose from the good decks. Moreover, the computer does not change the position of the decks once the game begins. It does not make you win or lose at random, or make you win or lose money based on the last card you picked.”
2. Seek reward instructions: Same as in the standard instructions, reward variant, except that the bold text is now “*The goal of the game is to win as much money as possible*.”
3. Avoid punishment instructions: Same as in the standard instructions, punishment variant, except that the bold text is now “*The goal of the game is to avoid losing as much money as possible*.”
4a. No-hint instructions, reward variant: “In front of you on the screen, there are four decks of cards: A′, B′, C′, and D′. When we begin the game, I want you to select one card at a time by clicking on a card from any of these decks. Sometimes you will win points, and sometimes you will lose points. You are absolutely free to switch from one deck to another at any time, and as often as you wish. You would not know when the game will end. Simply keep on playing until the computer stops. You will have $2,000 of credit, shown by the green bar, at the start of the game. Moreover, the computer does not change the position of the decks once the game begins. It does not make you lose at random, or make you lose money based on the last card you picked.”
4b. No-hint instructions, punishment variant: “In front of you on the screen, there are four decks of cards: E′, F′, G′, and H′. When we begin the game, I want you to select one card at a time by clicking on a card from any of these decks. Sometimes you will win points and sometimes you will lose points. You are absolutely free to switch from one deck to the other at any time, and as often as you wish. You would not know when the game will end. Simply keep on playing until the computer stops. You will have $2,000 of credit, shown by the green bar, at the start of the game. Moreover, the computer does not change the position of the decks once the game begins. It does not make you lose at random, or make you lose money based on the last card you picked.”
